# Intraosseous Lipoma of Calcaneus An Uncommon Cause of Heel Pain: A Case Report

**DOI:** 10.31729/jnma.8581

**Published:** 2024-05-31

**Authors:** Dilip Kumar Yadav, Ajay Kumar Yadav, Sujan Raj Paudel, Dilendra Yadav

**Affiliations:** 1 Department of Orthopaedic and Trauma Surgery, Scheer Memorial Adventist Hospital, Kavrepalanchok, Nepal; 2 Department of Internal Medicine, Kirtipur Hospital, Kathmandu, Nepal

**Keywords:** *bone cement*, *calcaneus*, *excision*, *intraosseous*, *lipoma*

## Abstract

Intraosseous lipoma of calcaneum is a rare cause of heel pain. Calcaneum is a typical site of involvement of IOL. There are only a few published articles regarding calcaneal intraosseous lipoma and one has been reported from Nepal. We report a case of 35 years female who presented with left heel pain for 1 year. The pain was gradually increasing in intensity and was severe enough to refrain her from activities of daily living. She was surgically operated with curettage and filling the defect with bone cement. There is no residual pain at 2.5 years follow up. We briefly review the postulated pathogenesis, clinical manifestations, diagnosis and various modalities of treatment of intraosseous lipoma. An orthopedic surgeon should have high degree of suspicion regarding the uncommon cause of heel pain and its possible management. When conservative methods do not relieve symptoms, surgical excision and filling the defect with bone cement provides long term relief.

## INTRODUCTION

One of the most frequent conditions seen in the orthopedics OPD (Outpatient Department) is heel discomfort. Plantar fasciitis and tendo Achilles tendinittis are the two conditions that cause heel discomfort the most frequently. The Intraosseous Lipoma (IOL) of Calcaneus is one of the unusual reasons of heel discomfort, though there are others as well. Intraosseous lipomas have been reported to occur throughout the skeleton with incidence of 8% in the calcaneus.^1^ As this condition is rare there is delay in diagnosis thus compromising the quality of life of patient. We hereby report a case of 35 years female who was diagnosed with intraosseous lipoma of calcaneus and was managed surgically. As there is paucity of literatures regarding IOL of calcaneum,we expect this article to be helpful in early diagnosis and intervention of the condition.

## CASE REPORT

A 35-year-old female presented to orthopedic OPD with complaints of left heel pain for 1 year. The pain was continuous pricking type, and aggravated by walking on uneven surface. The pain was gradually increasing in intensity and she had difficulty ambulating and performing activities of daily living for the past 3 months. There was no any history of trauma or twisting of her ankle and she had no any other comorbid conditions. The patient visited local health clinic for her symptoms where she was given some analgesics and advised for rest with no any further investigations. Her pain didn't subside and was gradually increasing so she presented to our hospital for further management. On physical examination, there was minimal swelling over lateral aspect of heel and tenderness over the base and lateral aspect of heel. There was no any evidence of skin changes or local rise of temperature, ankle instability, posterior heel pain (Tendo-achilles insertion). Ankle range of movements was full without any restrictions.

**Figure 1:**
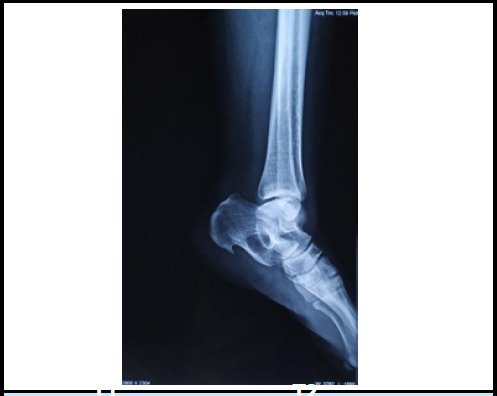
A lateral radiograph of ankle shows small cystic lesion in mid-calcaneus.

**Figure 2:**
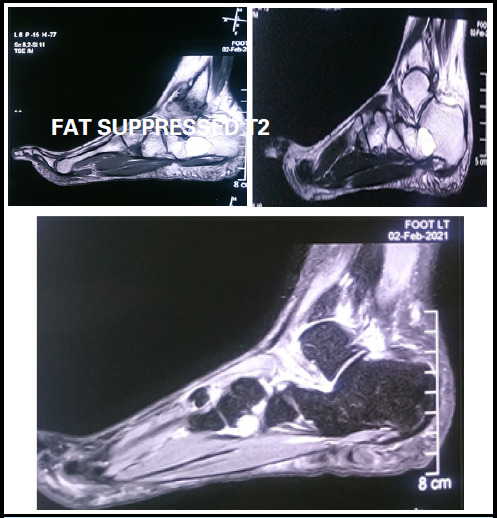
T1, T2 and Fat Suppressed T2 images (T1 showing high signal image,T2 showing intermediate signal image and fat suppressed T2 showing low signal image suggestive of intraosseous lipoma).

A plain radiograph of ankle in AP and lateral views were ordered which showed a well-defined cystic lesion in the mid-calcaneus (Figure 1). To confirm the lesion plain magnetic resonance imaging (MRI) of foot and ankle was advised which showed approximately 23.6 × 16.6 × 22.2 mm size well defined fat signal intensity lesion in mid calcaneus. The lesion had high signal in T1, intermediate signal in T2 and low signal in fat suppressed T2 weighted images (Figure 2) suggestive of intraosseous lipoma of calcaneus.

Hence, with all these workup, the diagnosis of intraosseous lipoma of calcaneus was made and surgical intervention was advised.

**Figure 3:**
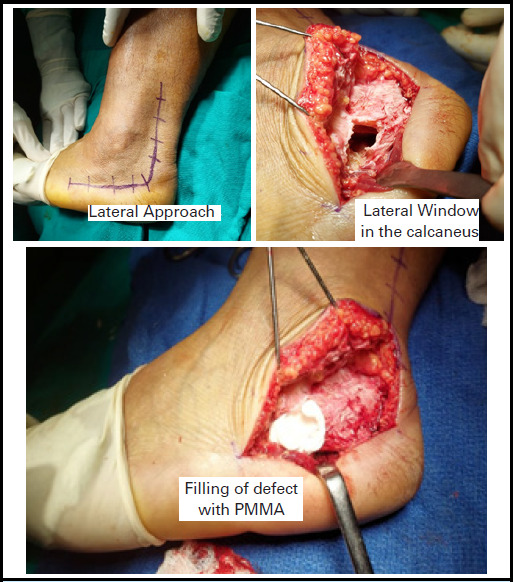
Intraoperative images showing lateral approach, lateral window in the calcaneus to remove the content and filling of the defect with PMMA.

**Figure 4:**
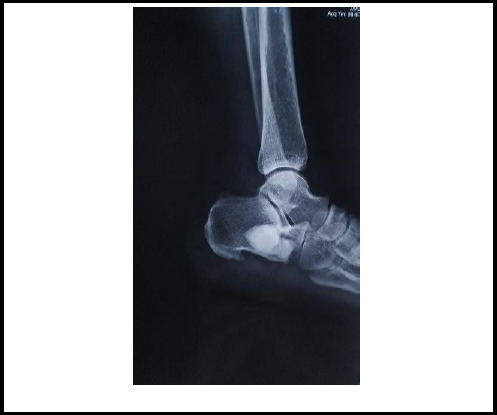
Immediate post-op showing defect filled with PMMA.

The calcaneus was approached laterally and the cyst was identified with C-arm. A bony window was made at lateral wall and the content was curetted which grossly showed soft cystic tissues with yellowish material. The cyst was removed adequately and washed thoroughly with povidine-iodine and Hydrogen peroxide solution. The void was washed and dried adequately and the defect was filled with polymethylmethacrylate (PMMA) cement (Figure 3). The subcutaneous tissue and skin was closed in layers. The patient was advised for weight wearing as tolerable for initial 2 weeks and then after full weight bearing. Post operatively X-ray done to confirm the adequate removal of lipoma of the defect with PMMA (Figure 4).

She was at regular follow up at our center, and was symptomatically improving. At about 2 months postsurgery she came to our OPD where she was totally pain free and was able to carry out her work (animal husbandry) comfortably. An X-ray of ankle joint was also repeated.

Postoperatively at 2.5 years follow-up, patient had no any residual pain and was able to perform her daily activities and work without any difficulties. An X-ray was done where there was no evidence of recurrence or any other changes.

## DISCUSSION

An uncommon cause of the frequent presentation of heel pain in an orthopedic clinic is an intraosseous lipoma of the calcaneus. A typical location for this benign tumor is the calcaneus. The cause of calcaneus intraosseous lipoma is uncertain. Three theories have been proposed regarding origin of intraosseous lipoma: (a) Traumatic origin and later fat degeneration (b) Infection, or osseous fat infarction with metaplasia and (c) third most studies think that intraosseous lipoma is a primary tumor of marrow fat.^2^ Intraosseous lipoma has no gender predilection and can occur at any age, often occurring in 4^th^ decade.^3^ In most cases intraosseous lipoma is an incidental finding while exploring the cause of heel pain. MRI is the best diagnostic tool with T1 (high intensity), T2 (shortening of signal) and STIR (suppression of fat) images confirming the fatty nature of the lesion. The study of lesions with CT or MRI due to its capacity to identify the fatty component in the lesion, has been proposed to avoid a biopsy of the lesion in order to confirm the diagnosis.^2^ However, MRI sometimes may not be able to differentiate between benign and malignant lesion.

Accordi ng to Mi lgrametal.'sclassifications,intraosseous lipomas are categorized into three stages based on the degree of fat necrosis: Stage 1- sharply limited lesion with homogenous fat content; Stage 2- Dominantly fatty lesions with central necrosis, calcification, or ossification, and Stage 3- A heterogeneous, fat containing lesion involving multiple necrotic areas, cystic transformations, sclerosis, or ossification in the wall.^4^

The majority of intraosseous lipomas can be treated conservatively with NSAIDs, cold compression, and the use of non-weight-bearing aids such a walking frame and silicone cast.^2^ Goto et al. recommended surgical management of intraosseous lipoma only when the lesion was painful, when there was a pathological fracture, when a histological diagnosis was required, or when it was necessary to reduce the danger of malignant transformation.^5^ Surgery is also indicated in the presence of pain resistant to conservative management.

Most often, the lipoma is debrided through a sufficient bone window, and then the defect is filled with autologous, allograft, bone hydroxyapatite, or PMMA cement. In actuality, when surgical intervention is required, curettage with bone grafting is the preferred course of action.^6^ In our case we did curettage of the lesion and filled the defect with PMMA due to its large defect and patient's demand.

Intraosseous lipoma of calcaneus has an excellent prognosis with almost negligible recurrence rate and malignant transformation. In spite of it, there are few cases of malignant transformation of preexisting bone lipoma in femur and tibia but never in calcaneus.^7^

Hence, one should consider IOL of calcaneum as possible cause of heel pain when dealing with pain around ankle. Our patient at subsequent follow up showed improvement of her symtoms and at 2.5 years follow up she had no any residual pain. However, long term outcomes and prognosis is yet to be explored.
